# Fine-tuning an aromatic ring-hydroxylating oxygenase to degrade high molecular weight polycyclic aromatic hydrocarbon

**DOI:** 10.1016/j.jbc.2024.107343

**Published:** 2024-05-04

**Authors:** Lihua Guo, Xingyu Ouyang, Weiwei Wang, Xiaoyu Qiu, Yi-Lei Zhao, Ping Xu, Hongzhi Tang

**Affiliations:** State Key Laboratory of Microbial Metabolism, and School of Life Sciences & Biotechnology, Shanghai Jiao Tong University, Shanghai, People’s Republic of China

**Keywords:** *Hydrogenibacillus*, aromatic ring-hydroxylating oxygenase, structure-guided rational enzyme engineering, polycyclic aromatic hydrocarbon, substrate specificity, catalytic efficiency

## Abstract

Rieske nonheme iron aromatic ring-hydroxylating oxygenases (RHOs) play pivotal roles in determining the substrate preferences of polycyclic aromatic hydrocarbon (PAH) degraders. However, their potential to degrade high molecular weight PAHs (HMW-PAHs) has been relatively unexplored. NarA2B2 is an RHO derived from a thermophilic *Hydrogenibacillus* sp. strain N12. In this study, we have identified four “hotspot” residues (V236, Y300, W316, and L375) that may hinder the catalytic capacity of NarA2B2 when it comes to HMW-PAHs. By employing structure-guided rational enzyme engineering, we successfully modified NarA2B2, resulting in NarA2B2 variants capable of catalyzing the degradation of six different types of HMW-PAHs, including pyrene, fluoranthene, chrysene, benzo[a]anthracene, benzo[b]fluoranthene, and benzo[a]pyrene. Three representative variants, NarA2B2^W316I^, NarA2B2^Y300F-W316I^, and NarA2B2^V236A-W316I-L375F^, not only maintain their abilities to degrade low-molecular-weight PAHs (LMW-PAHs) but also exhibited 2 to 4 times higher degradation efficiency for HMW-PAHs in comparison to another isozyme, NarAaAb. Computational analysis of the NarA2B2 variants predicts that these modifications alter the size and hydrophobicity of the active site pocket making it more suitable for HMW-PAHs. These findings provide a comprehensive understanding of the relationship between three-dimensional structure and functionality, thereby opening up possibilities for designing improved RHOs that can be more effectively used in the bioremediation of PAHs.

## Introduction

Polycyclic aromatic hydrocarbons (PAHs) are a common group of environmental pollutants noted for their persistence as well as carcinogenic and genotoxic properties (1). Leveraging the remarkable degradation capabilities of microorganisms, bioremediation has emerged as one of the most efficient and cost-effective methods for mitigating contamination by PAHs (2). Currently, research on microbial PAH degradation predominantly focuses on LMW-PAHs (3). In contrast, HMW-PAHs have reduced water solubility and increased toxicity (4), making them a critical focus for future studies of PAH degradation. The substrate specificity of PAH-degrader is dictated by aromatic ring-hydroxylating oxygenases (RHOs) under aerobic conditions (5). These enzymes are vital components of the degradation pathway. Regrettably, only a handful of RHOs have been reported thus far with the capability to degrade HMW-PAHs, including Nid (6, 7), Pdo (8), and Phn (9, 10) systems. Furthermore, the detailed degradation mechanisms of HMW-PAHs by these enzymes have also been less explored (11, 12). Comprehensive investigations into these mechanisms are imperative to advance our understanding and potentially enhance the bioremediation of HMW-PAHs.

Rational design is a classical enzyme engineering approach (13). This strategy relies on an understanding of the correspondence between protein structure and function to predict key amino acid residues that influence the properties of the enzyme (14). Subsequently, these crucial amino acid residues are replaced through site-directed mutagenesis techniques to achieve the desired effects (13, 14). This strategy has successfully fine-tuned various aspects of proteins, such as substrate specificity, catalytic efficiency, thermostability, and enantioselectivity (15, 16). For instance, the rational design of cumene dioxygenase from *Pseudomonas fluorescens* IP01 resulted in a 16-fold increase in enzyme activity and the formation of new products and enantiomers (17). Through structure-based computational enzyme design, the aspartase from *Bacillus* sp. YM55-1 demonstrated remarkable substrate tolerance, accommodating concentrations up to 300 g/L. Additionally, it achieved impressive conversion rates of up to 99%, a β-regioselectivity surpassing 99%, and a product enantiomeric excess exceeding 99% (18). This strategy of structure-based rational design has evolved into a potent tool, greatly improving the tailoring of biocatalysts to meet distinct industrial or research requirements.

Herein, we explored the catalytic properties and mechanisms of NarA2B2 (19) and NarAaAb (20) from the thermophilic *Hydrogenibacillus* sp. strain N12. Through protein structure prediction and molecular docking, we identified the “hotspot” residues that restrict NarA2B2’s catalysis of HMW-PAHs. Subsequently, we employed structure-guided rational enzyme engineering to modify NarA2B2. The modified NarA2B2 had enhanced degradation capabilities for HMW-PAHs, which catalyzed the degradation of six different types of HMW-PAHs with significantly higher catalytic efficiency than NarAaAb. Our computational biology analysis revealed that the ability of NarA2B2 variants to degrade HMW-PAHs was attributed to an enlarged active site pocket and increased hydrophobicity, facilitating the entry of HMW-PAHs. This in-depth exploration of catalytic characteristics and mechanisms of RHOs enhances our understanding of HMW-PAH biodegradation. Furthermore, the application of structure-guided rational enzyme engineering allows for the novel development of more proficient RHOs, holding substantial promise in the field of bioremediation and environmental cleanup.

## Results

### Catalytic characteristics of NarAaAb and NarA2B2

NarA2B2 and NarAaAb were two RHOs identified from *Hydrogenibacillus* sp. N12. The α subunits of NarA2B2 and NarAaAb, namely, NarA2 and NarAa, shared a moderate sequence similarity of approximately 45.6% (Fig. S1). Phylogenetic tree analysis categorized NarAaAb and NarA2B2 within the type V RHOs (21) (Fig. S1). Both NarA2B2 and NarAaAb demonstrated catalytic functions, necessitating the transfer of electrons through a [3Fe-4S]-type ferredoxin and a glutathione reductase-type reductase.

**Figure 1:**
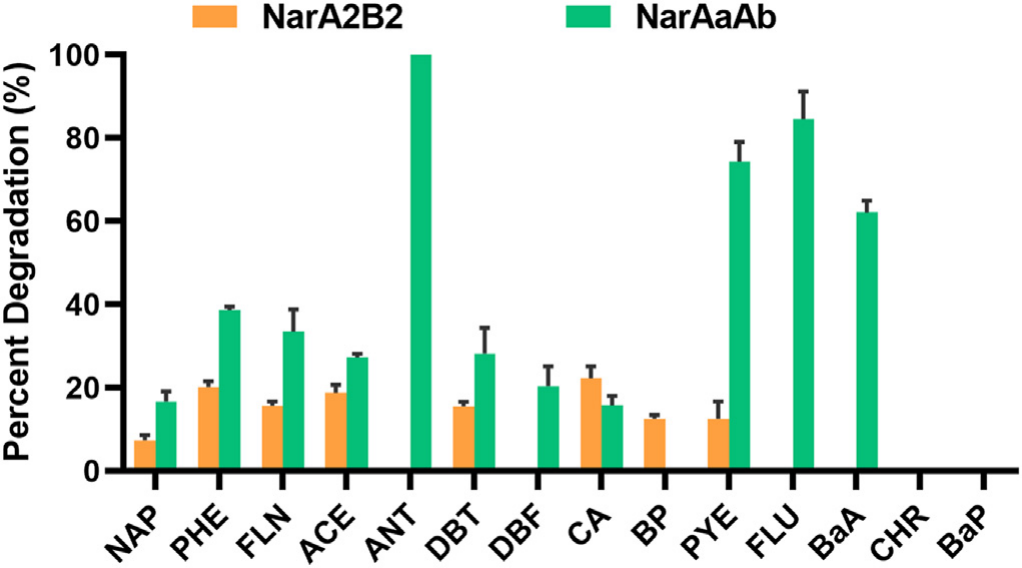
**The degradation percentages of PAHs and derivatives by NarAaAb and NarA2B2 within the whole cells at 24 h.** The green column represents *E. coli* BL21(DE3) containing pET28a-*narAaAb* and pACYCDuet-*phdCD*, and the *orange* column represents *E. coli* BL21(DE3) containing pETDuet-*narA2*-Ter-*narB2* and pACYCDuet-*phtAcAd*. NAP, naphthalene (50 mg/L); PHE, phenanthrene (50 mg/L); FLN, fluorene (50 mg/L); ACE, acenaphthene (50 mg/L); ANT, anthracene (40 mg/L); DBT, dibenzothiophene (50 mg/L); DBF, dibenzofuran (50 mg/L); CA, carbazole (50 mg/L); BP, biphenyl (50 mg/L); PYE, pyrene (10 mg/L); FLU, fluoranthene (10 mg/L); BaA, benzo[a]anthracene (10 mg/L); CHR, chrysene (10 mg/L); BaP, benzo[a]pyrene (10 mg/L). The concentration in parentheses is the initial concentration. HMW-PAHs: PYE, FLU, BaA, CHR, and BaP.

However, there were distinct differences in substrate specificity between the two enzymes. NarAaAb demonstrated a broader substrate spectrum compared to NarA2B2. NarAaAb exhibited efficient degradation capabilities towards a range of LMW-PAHs, including naphthalene (NAP), phenanthrene (PHE), dibenzothiophene (DBT), fluorene (FLN), acenaphthene (ACE), carbazole (CA), anthracene (ANT), and dibenzofuran (DBF). Moreover, it demonstrated proficiency in degrading three types of HMW-PAHs, namely pyrene (PYE), fluoranthene (FLU), and benzo[a]anthracene (BaA) (Figs. 1 and S2). In contrast, NarA2B2 only degraded LMW-PAHs and pyrene (19). When assessing catalytic activity within whole cells, NarAaAb exhibited significantly higher degradation efficiency for other substrates compared to NarA2B2, except for CA (Fig. 1). NarAaAb achieved the highest degradation percentage for ANT, reaching 100% at an initial concentration of 40 mg/L, while exhibiting the lowest degradation percentage for CA, at only 16.5% (Fig. 1). Additionally, NarAaAb had enhanced degradation capacity for HMW-PAHs, with degradation percentages of 85.7%, 75.0%, and 62.5% for FLU, PYE, and BaA, respectively, at initial concentrations of 10 mg/L (Fig. 1). Conversely, NarA2B2 could only degrade PYE, with a degradation percentage one-sixth that of NarAaAb (Fig. 1).

Furthermore, the metabolites of PAHs and their derivatives catalyzed by NarA2B2 and NarAaAb were detected through GC-MS, aiming to elucidate the catalytic properties of these RHOs (Fig. S3 and Table S1). Generally, NarA2B2 and NarAaAb exhibited dioxygenase activity in catalyzing PAHs and their derivatives, but they also function as monooxygenases in catalyzing ACE and FLN (Fig. S3). And they displayed different preferences for catalytic sites of specific substrates (Fig. S3). For instance, the degradation characteristics of NAP, FLN, ACE, and PYE by the two enzymes were consistent overall. However, in the catalysis of PHE and DBT, NarAaAb and NarA2B2 possess different catalytic properties. In the case of DBT, NarA2B2 tended to produce dibenzothiophene-S-oxide, whereas NarAaAb followed the Kodama pathway (22), introducing oxygen to the C-1 and C-2 positions of DBT (Fig. S3).

Therefore, we can confirm that the substrate specificity and catalytic properties of the two RHOs, NarA2B2 and NarAaAb, in strain N12 exhibit significant differences. NarAaAb was capable of catalyzing a wider range of HMW-PAHs.

### Structural specificity of NarAaAb and NarA2B2

We analyzed the three-dimensional structures of both enzymes to elucidate the underlying causes of their notable differences in substrate specificity and catalytic properties.

**Figure 2:**
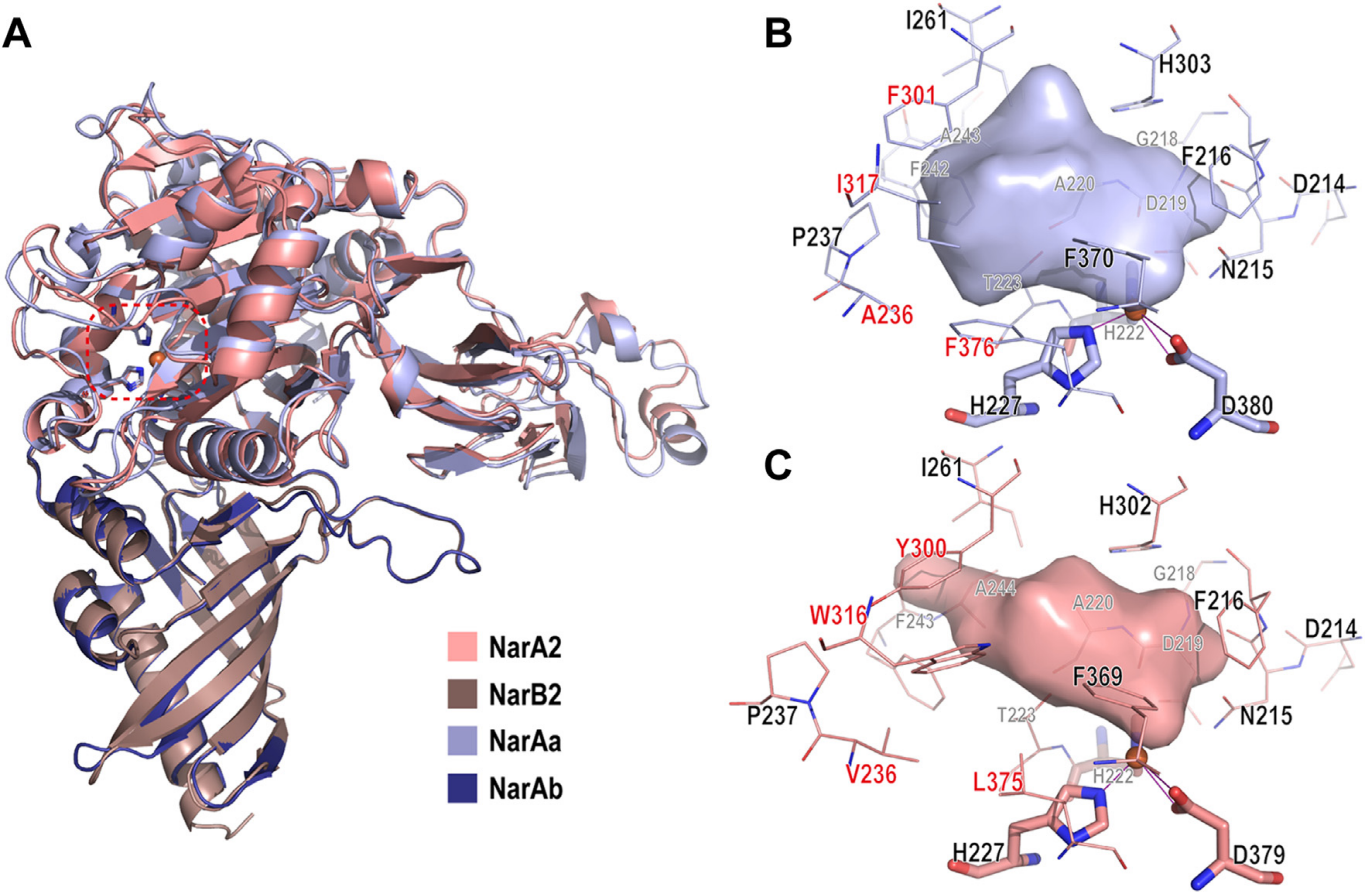
**Predicted structures and active sites of NarAaAb and NarA2B2.***A*, structures of NarAaAb and NarA2B2 generated by AlphaFold2 Multimer, display distinct chains in various colors, with the iron atom positioned based on homologous structures, the active site was highlighted by a *dashed line border*. *B*, active site of NarAa and (*C*) NarA2, with pocket surface predicted by POCASA 1.1. Thin sticks represent residues near the docked substrate (within 4 Å), and normal sticks represent residues that coordinate with iron. Important residues that vary between NarAaAb and NarA2B2 in the active site are marked in *red*.

The structures of NarAa and NarA2 exhibit sequence identities of 65.8% and 51.7%, respectively, with the naphthalene dioxygenase large subunit (PDB ID 2B1X) (Fig. S4). Subsequently, we employed the highest-scoring models predicted by AlphaFold2 Multimer for NarAaAb and NarA2B2 for analysis, revealing considerable similarities in secondary structure. This is evidenced by the low Root Mean Square Deviation values: 0.807 Å for NarAa relative to NarA2, and 0.343 Å for NarAb relative to NarB2 (Fig. 2*A*). Concerning the iron-center active site, both structures are identified as nonheme iron oxygenases, incorporating the typical 2-His-1-carboxylate facial triad (23). The active sites of NarAa and NarA2, represented by H227-H222-D380 and H227-H222-D379 sequences, respectively, are vital for dioxygen activation.

Structural differences between NarAaAb and NarA2B2 suggest varying substrate preferences. For instance, the active site pocket in NarAa has a volume of 89 Å^3^, compared to 82 Å^3^ in NarA2, each presenting different shapes as predicted by POCASA 1.1 using default parameters (Fig. 2, *B* and *C*). The residues surrounding the active site influence substrate specificity. Specifically, in NarAa, ^NarAa^F301, ^NarAa^I317, ^NarAa^A236, and ^NarAa^F376 differ from NarA2’s corresponding residues ^NarA2^Y300, ^NarA2^W316, ^NarA2^V236, and ^NarA2^L375 (Fig. 2, *B* and *C*). These unique residues in NarAa, particularly ^NarAa^F301, ^NarAa^I317, ^NarAa^A236, and ^NarAa^F376 may be essential in degrading HMW-PAHs. Notably, ^NarA2^W316 introduces significant spatial constraints, reducing the volume of NarA2 and resulting in a narrower shape. Such structural specificity affords insight into NarAa’s preference for HMW-PAHs, in contrast to NarA2.

### Verification and computational analysis of the key residues of NarAa

**Figure 3:**
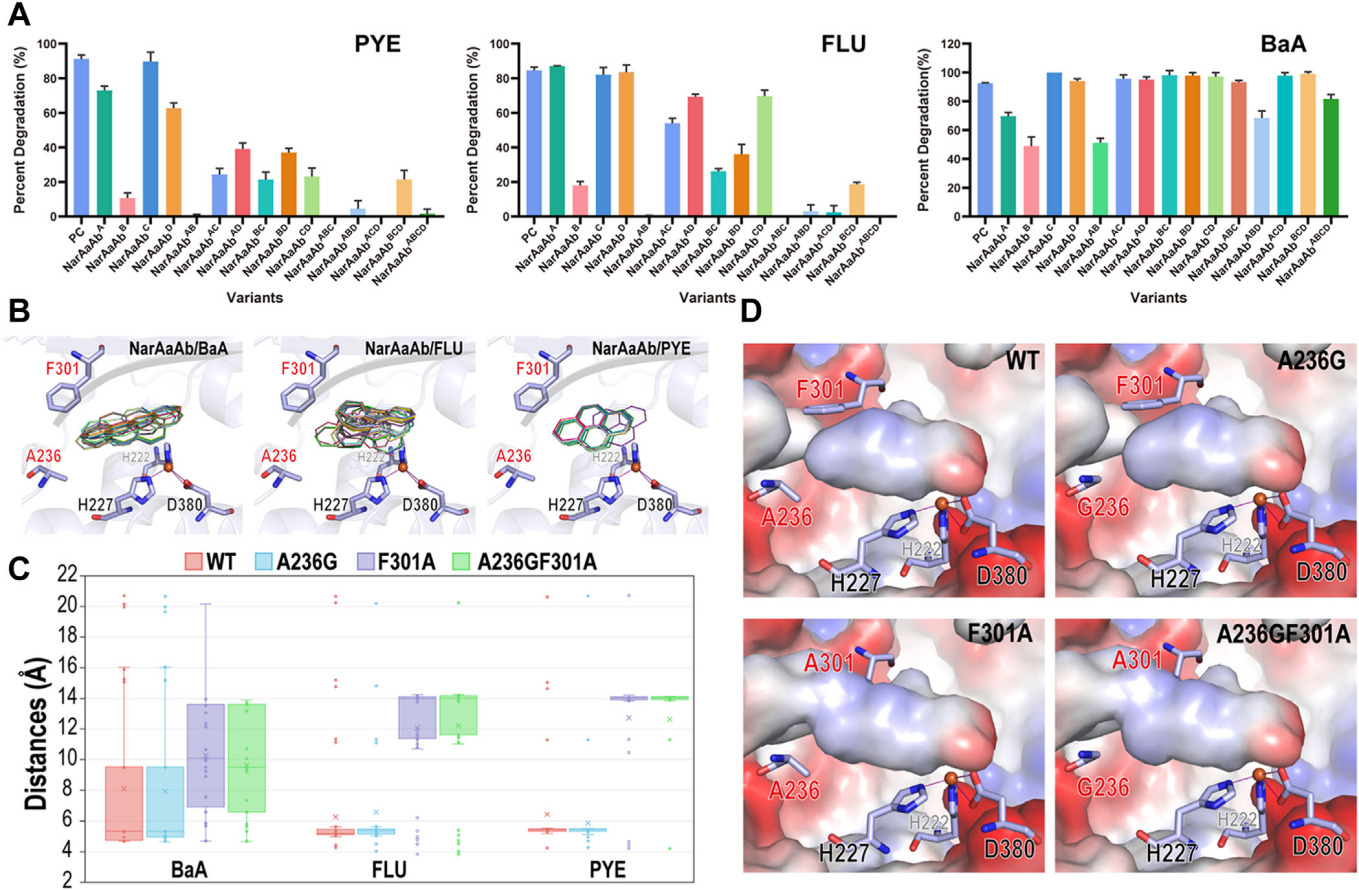
**The degradation of HMW-PAHs by NarAaAb variants and analysis of key residues in NarAa.***A*, the degradation of PYE, FLU, and BaA by NarAaAb variants. PYE: pyrene; FLU: fluoranthene; BaA: benzo[a]anthracene. PC: *E. coli* BL21(DE3) containing pET28a-*narAaAb* and pACYCDuet-*phdCD* without mutations; NarAaAb^A^: NarAaAb^A236G^; NarAaAb^B^: NarAaAb^F301A^; NarAaAb^C^: NarAaAb^I317A^; NarAaAb^D^: NarAaAb^F376A^; NarAaAb^AB^: NarAaAb^A236G-F301A^; NarAaAb^AC^: NarAaAb^A236G-I317A^; NarAaAb^AD^: NarAaAb^A236G-F376A^; NarAaAb^BC^: NarAaAb^F301A-I317A^; NarAaAb^BD^: NarAaAb^F301A-F376A^; NarAaAb^AD^: NarAaAb^A236G-F376A^; NarAaAb^ABC^: NarAaAb^A236G-F301A-I317A^; NarAaAb^ABD^: NarAaAb^A236G-F301A-F376A^; NarAaAb^ACD^: NarAaAb^A236G-I317A-F376A^; NarAaAb^BCD^: NarAaAb^F301A-I317A-F376A^; NarAaAb^ABCD^: NarAaAb^A236G-F301A-I317A-F376A^. *B*, the docking results of WT of NarAa with three representative substrates. Multiple docking runs of the substrate are depicted by thin sticks, while residues of the mutation sites 236 and 301, as well as the iron coordination residues, are displayed with regular sticks. *C*, a box plot illustrating the distances between the center-of-mass of docked substrates and the iron atom. Molecular docking was performed with 100 sampling runs. *D*, the electrostatic potential surface of different variants of NarAa, depicted without substrates, with negative potential in *red* and positive potential in *blue*.

To identify the essential residues in NarAa associated with HMW-PAHs catalysis, we introduced point mutations and assessed their impact on HMW-PAHs degradation using the resting cell biotransformation method (Fig. 3*A*). The assays were consistent with the predictions, as many variants showed reduced catalytic efficiency towards HMW-PAHs. Among them, the most pronounced effects were observed for NarAaAb^F301A^ and NarAaAb^A236G-F301A^. NarAaAb^F301A^ significantly decreased the degradation of PYE and FLU by 80.5% and 66.5%, respectively (Fig. 3*A*). NarAaAb^A236G-F301A^ lost the ability to degrade both FLU and PYE. Similarly, variants like NarAaAb^A236G-F301A-I317A^ and NarAaAb^A236G-I317A-F376A^ also negatively affected the degradation of FLU and PYE (Fig. 3*A*). In contrast to degradation effects on FLU and PYE, fewer variants showed inhibition on the degradation of BaA, with NarAaAb^A236G^, NarAaAb^F301A^, and NarAaAb^A236G-F301A^ reducing degradation by 22.9%, 43.7%, and 41.3%, respectively. However, NarAaAb^A236G-F301A-I317A-F376A^ and NarAaAb^A236G-F301A-F376A^ exhibited milder reductions in the catalytic efficiency of BaA, with degradation percentages decreasing by 11.1% and 24.2%, respectively (Fig. 3*A*). The experimental results underscore the pivotal role of these four amino acids in NarAaAb’s catalytic activity toward HMW-PAHs.

Substituting residues surrounding the NarAa’s active site highlighted the importance of residues A236 and F301 in maintaining the active site pocket’s structure and substrate specificity. Based on docking results for the wild type (WT), three representative substrates, BaA, FLU, and PYE, consistently maintain their positions within the active site (Fig. 3*B*). During multiple docking tests on the WT and variants, the distances between the center of mass of the substrate and the catalytic iron were determined, as depicted in Figure 3*C*. Upon introducing the mutation F301A, the observed distances within the enzyme increased significantly, suggesting the enlargement of the active site or the formation of a new tunnel (Fig. 3*D*). The mutation A236G alone has minimal impact on the enzyme’s activity. However, when combined with mutation F301A, it led to a widened tunnel in NarAaAb^A236G-F301A^, which may inadvertently reduce the accessibility of HMW-PAHs to the active site of the enzyme (Fig. 3*D*). In addition, we can infer that a decrease in π-π interaction between the substrate and NarAaAb^F301A^ limited substrate stability.

### Modification of NarA2B2 to degrade HMW-PAHs

**Figure 4:**
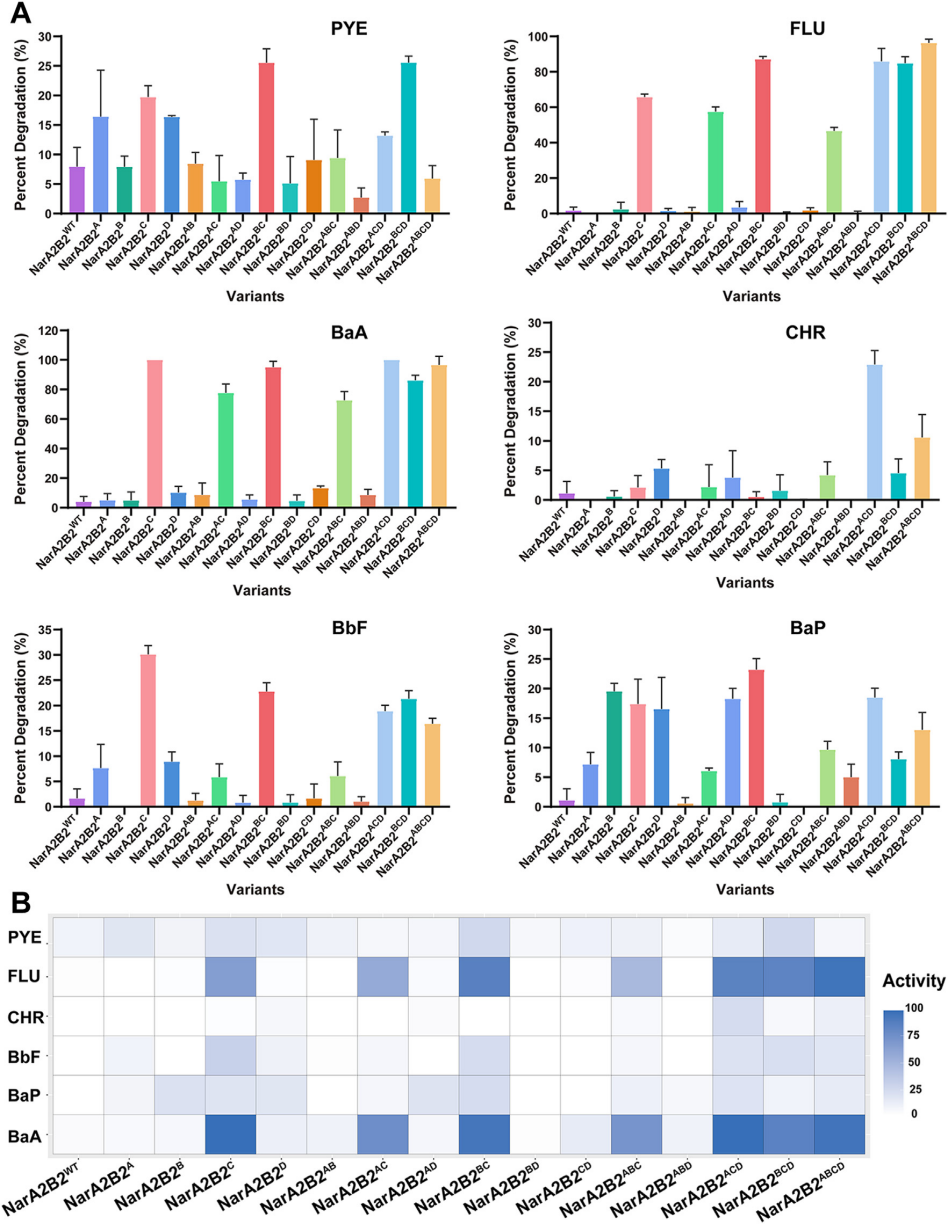
**The degradation of HMW-PAHs by the resting cells which expressed NarA2B2 and PhtAcAd.***A*, the degradation percentages of HMW-PAHs by *E. coli* BL21(DE3) containing pETDuet-*narA2*-Ter-*narB2* and pACYCDuet-*phtAcAd* at 24 h. *B*, the heat map of degradation percentages of HMW-PAHs by NarA2B2 variants. PYE: pyrene; FLU: fluoranthene; BaA: benzo[a]anthracene; CHR: chrysene; BbF: benzo[b]fluoranthene; BaP: benzo[a]pyrene. NarA2B2^WT^: the WT of NarA2B2; NarA2B2^A^: NarA2B2^V236A^; NarA2B2^B^: NarA2B2^Y300F^; NarA2B2^C^: NarA2B2^W316I^; NarA2B2^D^: NarA2B2^L375F^; NarA2B2^AB^: NarA2B2^V236A-Y300F^; NarA2B2^AC^: NarA2B2^V236A-W316I^; NarA2B2^AD^: NarA2B2^V236A-L375F^; NarA2B2^BC^: NarA2B2^Y300F-W316I^; NarA2B2^BD^: NarA2B2^Y300F-L375F^; NarA2B2^CD^: NarA2B2^W316I-L375F^; NarA2B2^ABC^: NarA2B2^V236A-Y300F-W316I^; NarA2B2^ABD^: NarA2B2^V236A-Y300F-L375F^; NarA2B2^ACD^: NarA2B2^V236A-W316I-L375F^; NarA2B2^BCD^: NarA2B2^Y300F-W316I-L375F^; NarA2B2^ABCD^: NarA2B2^V236A-Y300F-W316I-L375F^.

Inspired by the determination of the essential residues of NarAa in degrading HMW-PAHs, we endeavored to confer the capabilities of catalyzing HMW-PAHs to NarA2B2 through structure-guided rational enzyme engineering. We utilized two methods, “resting cell biotransformation” and “enzyme assay”, to determine the catalytic activity of the modified NarA2B2 towards HMW-PAHs. It was astonishing that, upon replacing the corresponding amino acid residues in NarA2 with those identified as essential in NarAa, many substitutions enhanced the ability to degrade multiple HMW-PAHs (Fig. 4).

When assessing the degradation of HMW-PAHs by the expressed NarA2B2 variant in resting cells, we observed NarA2B2^W316I^, NarA2B2^Y300F-W316I^, and NarA2B2^V236A-W316I-L375F^ exhibited significant degradation impacts. NarA2B2^W316I^ could degrade FLU, BaA, benzo[b]fluoranthene (BbF), and benzo[a]pyrene (BaP), with degradation percentages of 65.8%, 100%, 30.1%, and 17.4%, respectively. In addition, compared to the WT of NarA2B2, NarA2B2^W316I^ exhibited an 11.8% increase in the degradation percentage of PYE. NarA2B2^Y300F-W316I^ demonstrated a similar impact to NarA2B2^W316I^, with a more pronounced degradation of FLU, PYE, and BaP, resulting in degradation percentages of 87.1%, 25.5%, and 23.2%, respectively. Remarkably, NarA2B2^V236A-W316I-L375F^ demonstrated distinct catalytic activity towards all six types of HMW-PAHs, with degradation percentages for PYE, FLU, BaA, chrysene (CHR), BbF, and BaP being 13.2%, 85.9%, 100%, 22.9%, 18.9%, and 18.5%, respectively. Other variants also had proficiency to catalyze HMW-PAHs, but their degradation efficacy was weaker, or they showed limited degradation towards a subset of HMW-PAHs, such as NarA2B2^V236A-Y300F-W316I-L375F^ and NarA2B2^Y300F-W316I-L375F^. Overall, structure-guided rational enzyme engineering enabled NarA2B2 to degrade a variety of HMW-PAHs (Fig. 4, *A* and *B*).

Given the remarkable degradation capabilities of NarA2B2^W316I^, NarA2B2^Y300F-W316I^, and NarA2B2^V236A-W316I-L375F^ on HMW-PAHs, we undertook purification of these variants to examine their catalytic properties toward PAHs. Prior to this, we explored the optimal catalytic conditions for NarA2B2 and NarAaAb. The optimal catalytic temperature for purified NarAaAb was found to be 30 °C when PhtAcAd was used as the electron transport protein, with significantly limited activity observed at 42 °C. In contrast, purified NarA2B2 exhibited a higher optimal catalytic temperature of 37 °C and maintained a high catalytic efficiency even at higher temperatures (Fig. S5). NarA2B2 and NarAaAb exhibit optimal catalytic efficiency at pH seven and displayed no catalytic activity in acidic environments when adapted to PhtAcAd (Fig. S5).

**Figure 5:**
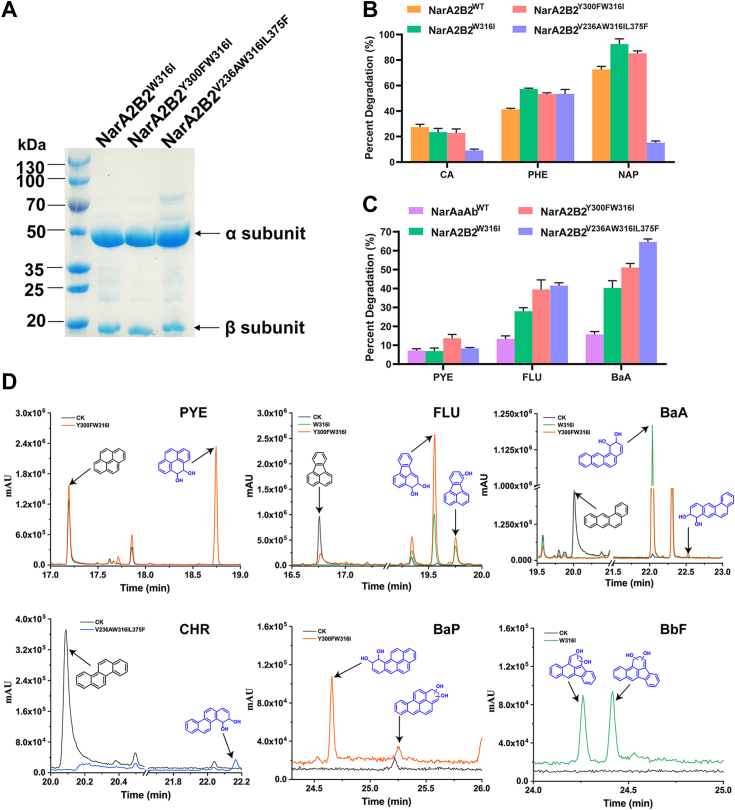
**The degradation of PAHs catalyzed by purified NarA2B2 variants.***A*, the purification of NarA2B2 variants by Ni-NTA affinity chromatography. *B*, the degradation of LMW-PAHs by purified NarA2B2 variants. *C*, the degradation of HMW-PAHs by NarA2B2 variants. *D*, GC-MS profile of HMW-PAHs catalyzed by purified NarA2B2 variants. The *black line* represented that the mixture of enzyme assay did not contain the enzyme, the *orange line* represented that the mixture of enzyme assay contained the NarA2B2^Y300FW316I^ and PhtAcAd, the *green line* represented that the mixture of enzyme assay contained NarA2B2^W316I^ and PhtAcAd, the *blue line* represented that the mixture of enzyme assay contained NarA2B2^V236A-W316I-L375F^ and PhtAcAd.

The proteins with high purity were obtained through Ni-NTA affinity chromatography (Fig. 5*A*). The purified protein was utilized for the degradation of HMW-PAHs, with the objective of investigating both their catalytic properties and the underlying mechanism. Notably, NarA2B2^W316I^, NarA2B2^Y300F-W316I^, and NarA2B2^V236A-W316I-L375F^ demonstrated catalytic activity towards PAHs containing between two and five rings under the optimal catalytic conditions (Fig. 5, *B–D*). NarA2B2^W316I^ and NarA2B2^Y300F-W316I^ exhibited similar activity when degrading LMW-PAHs, while NarA2B2^V236A-W316I-L375F^ exhibited lower catalytic activity towards NAP and CA (Fig. 5*B*). In contrast to NarAaAb, NarA2B2 variants showed significantly higher enzyme activity against HMW-PAHs (Fig. 5*C*). Interestingly, NarA2B2^V236A-W316I-L375F^ demonstrated the highest activity when catalyzing FLU and BaA (Fig. 5*C*). Compared to the WT of NarAaAb, NarA2B2^Y300F-W316I^ exhibited a 6.5% increase in the degradation percentage of PYE. Furthermore, NarA2B2^W316I^, NarA2B2^Y300F-W316I^, and NarA2B2^V236A-W316I-L375F^ demonstrated significantly enhanced degradation efficiencies for FLU and BaA. Specifically, their degradation efficiencies for FLU were 2.1, 2.96, and 3.12 times higher, while their degradation efficiencies for BaA were improved by 2.56, 3.24, and 4.10 times compared to the WT of NarAaAb (Fig. 5*C*).

**Figure 6:**
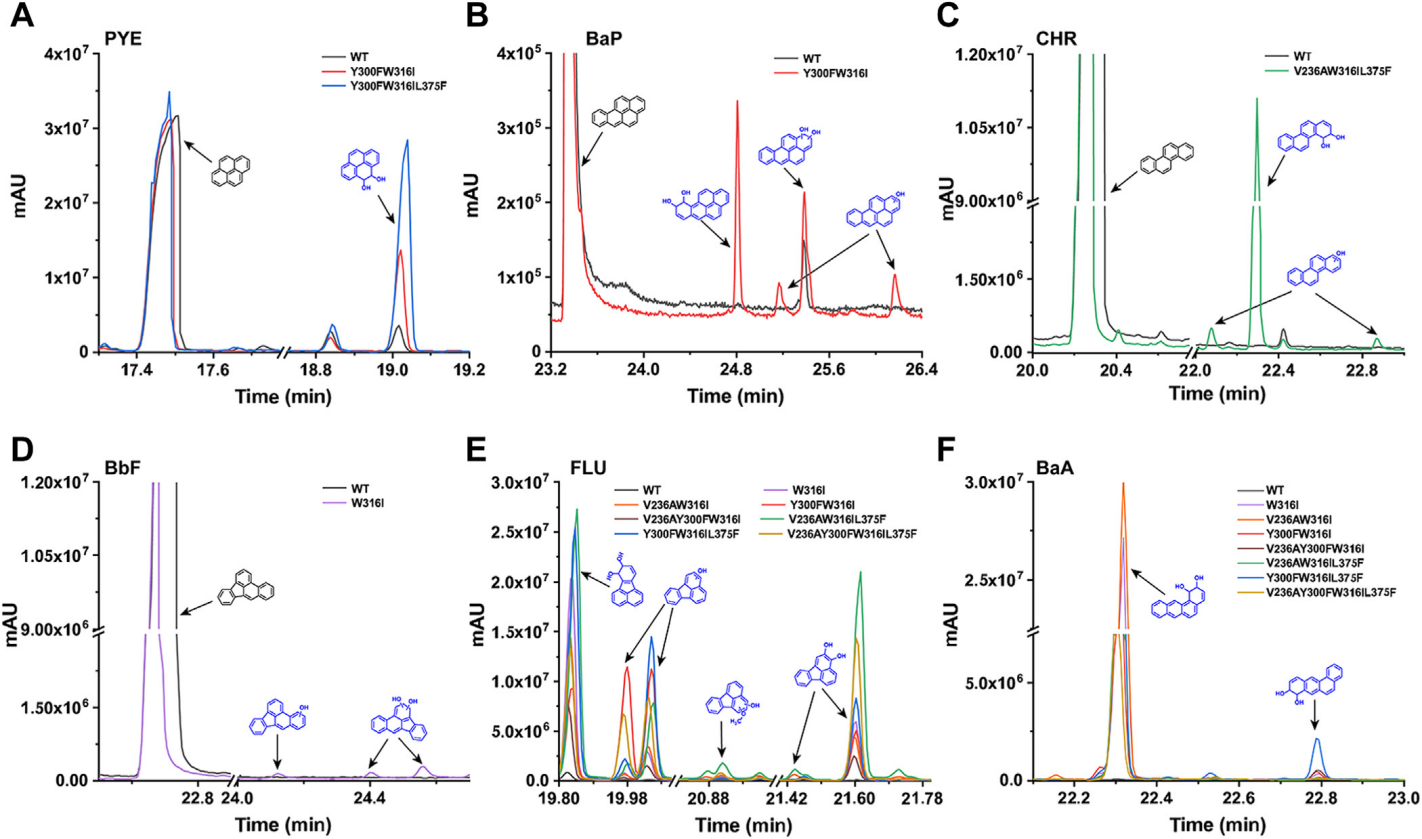
**GC-MS profile of HMW-PAHs catalyzed within the whole cells which expressed NarA2B2 variants and PhtAcAd.***A*, PYE; (*B*) BaP; (*C*) CHR; (*D*) BbF; (*E*) FLU; (*F*) BaA. The hydrogen ions of the hydroxyl groups in all metabolites are replaced by trimethylsilyl groups.

To further elucidate the catalytic properties of the modified NarA2B2 towards HMW-PAHs, we employed GC-MS to analyze the catalytic products of HMW-PAHs by both the expressed NarA2B2 variant in resting cells and the purified NarA2B2 variants protein (Table 1). When NarA2B2^Y300F-W316I^ acted on PYE, only PYE-4,5-dihydrodiol was detected, suggesting that this variant increased the degradation percentage of PYE without affecting its catalytic positions (involving adding two oxygen atoms to the C-4 and C-5 positions) (Figs. 5*D*, 6 and S3). Similar to NarAaAb, NarA2B2^W316I^ and NarA2B2^Y300F-W316I^ catalyzed FLU at the C-2 and C-3 positions, resulting in the detection of FLU-2,3-dihydrodiol and a specific monohydroxy-FLU in their degradation samples (Figs. 5*D*, 6 and S3). In comparison to NarAaAb, NarA2B2 variants exhibited a distinct preference in the catalytic sites for BaA. NarA2B2^W316I^ and NarA2B2^Y300F-W316I^ catalyzed BaA at the C-1 and C-2 positions, producing BaA-1,2-dihydrodiol (Figs. 5*D*, 6 and S3). NarA2B2^Y300F-W316I^, however, transformed BaA into BaA-8,9-dihydrodiol, while the yield of BaA-8,9-dihydrodiol was relatively low compared to BaA-1,2-dihydrodiol. Finally, NarA2B2^V236A-W316I-L375F^ catalyzed CHR by introducing two oxygen atoms to the C-3 and C-4 positions, forming CHR-3,4-dihydrodiol (Figs. 5*D* and 6). For BaP catalysis by NarA2B2^Y300F-W316I^, the primary product was BaP-9,10-dihydrodiol, alongside a dihydrodiol with an unknown structure (Figs. 5*D* and 6). In the case of BbF degradation by NarA2B2^W316I^, two distinct BbF-dihydrodiol products were identified (Figs. 5*D* and 6), although determining the exact locations of the hydroxyl groups was challenging due to limited information. The lack of suitable standards prevented rigorous assessment of the levels of products, but some appear to be at levels of 6 to 7%, based on a 1,2-dihydroxynaphthalene internal standard (Table S2).

**Table 1:** GC-MS analysis of HMW-PAHs metabolites produced by NarA2B2 variants

Substrate	Metabolites	Structure	Retention time (min)	Mass spectral characteristics of product (ion abundances)
PYE	PYE-4,5-dihydrodiol		18.744	147 (100), 73 (70), 290 (60), 202 (35), 291 (34), 189 (27), 380 (22)
FLU	FLU-2,3-dihydrodiol		19.559	73 (100), 147 (62), 190 (54), 380 (46), 218 (33), 189 (30), 202 (28)
	monohydroxy-FLU	__	19.749	290 (100), 275 (65), 189 (40), 73 (28), 291 (27), 200 (23), 215 (18)
BaA	BaA-1,2-dihydrodiol		22.036	73 (100), 191 (61), 147 (28), 303 (27), 215 (24), 216 (17), 228 (14)
	BaA-8,9-dihydrodiol		22.527	73 (100), 191 (51), 147 (28), 303 (25), 215 (23), 207 (21), 75 (19)
CHR	CHR-3,4-dihydrodiol		22.169	73 (100), 191 (59), 147 (35), 215 (28), 75 (25), 207 (21), 406 (15)
BaP	BaP-9,10-dihydrodiol		24.658	73 (100), 191 (53), 239 (31), 147 (27), 327 (24), 252 (21), 207 (19)
	BaP-dihydrodiol	__	25.253	73 (100), 207 (71), 191 (53), 281 (29), 75 (29), 147 (27), 133 (21)
BbF	BbF-dihydrodiol	__	24.260	73 (100), 191 (55), 147 (24), 239 (22), 75 (19), 207 (18), 240 (18)
	BbF-dihydrodiol	__	24.416	73 (100), 191 (78), 147 (25), 207 (20), 239 (19), 240 (18), 252 (16)

The hydrogen ions of the hydroxyl groups in all metabolites are replaced by trimethylsilyl groups.

The chemical structures of all metabolites of HMW-PAHs were determined by comparison with published data.

The metabolites whose structure was not determined were indicated by “__”.

### Substrate orientations for catalysis in modified NarA2B2

**Figure 7:**
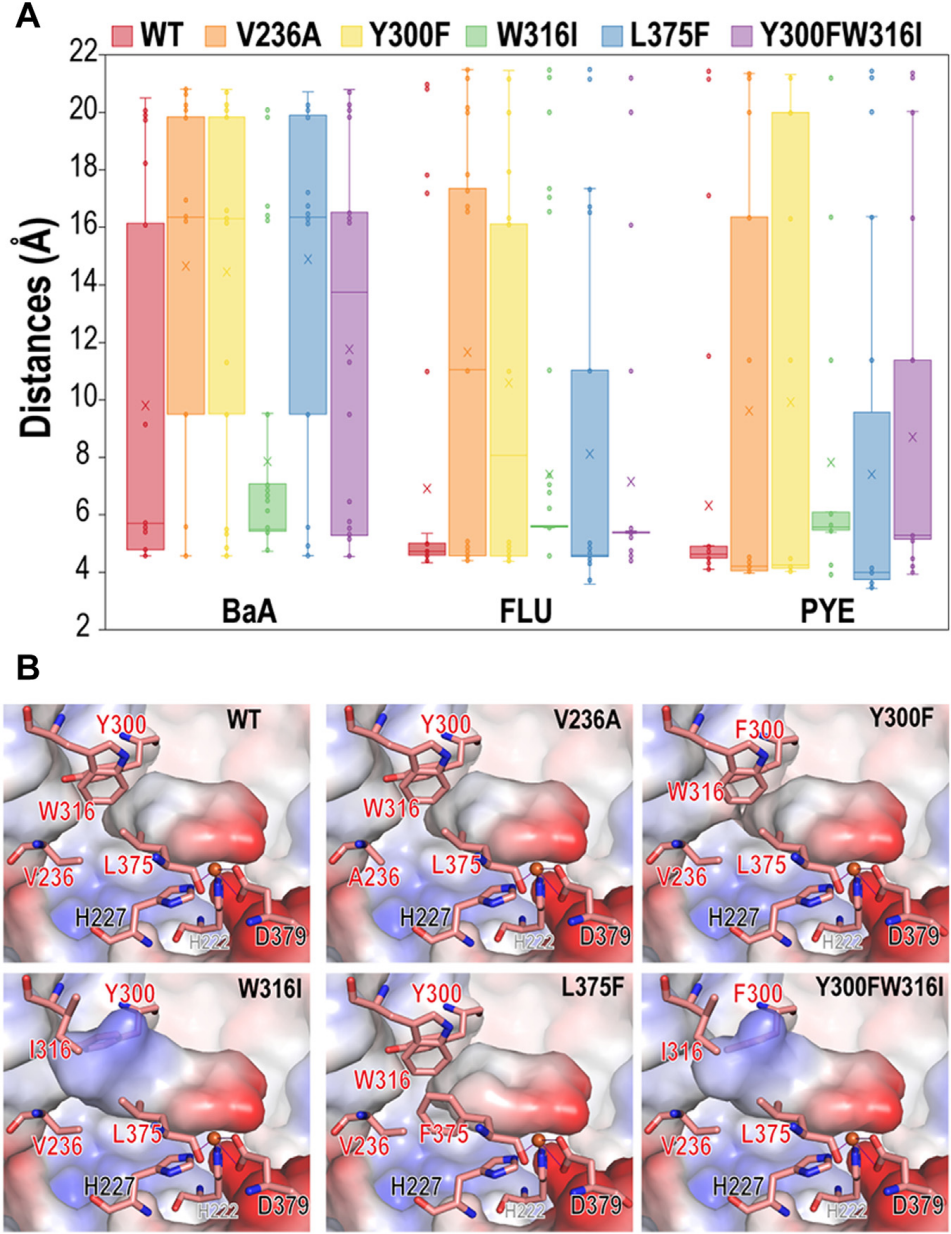
**Structural Analysis of NarA2.***A*, box plot showing the distances between the center-of-mass of docked substrates and the iron atom. Molecular docking was conducted on NarA2 (WT and variants) with three representative substrates, using 100 sampling runs. *B*, the electrostatic potential surface of different variants of NarA2, depicted without substrates, with negative potential in *red* and positive potential in *blue*.

Through comparisons of the structures and substrate profiles of NarAaAb and NarA2B2, NarA2 was modified to acquire the ability to degrade HMW-PAHs, including FLU and BaP, *etc.* Multiple repetitions of molecular docking indicated variations in distance (Fig. 7*A*). While the distances between the substrates and the iron atom in the WT generally appeared very close, these distances were often too narrow to properly accommodate the dioxygen molecule, as seen with FLU and PYE in WT in Figure 7*A*. Conversely, due to its longer, linear molecular structure, BaA was occasionally only able to dock on the protein’s surface, as shown in WT in Figure 7*A*, resulting in greater variability in its docking distances.

Docking studies suggested that replacing larger residues with smaller ones could increase the active site pocket’s capacity to accommodate substrates. The surface views of variants also indicate that the shape and volume of the active site pocket are altered depending on the surrounding residues. NarA2B2^W316I^ enlarges the pocket without causing substrate leakage (*i.e.*, no significant novel tunnel is established as in NarAa^F301A^). Combining W316I with Y300F not only optimizes the shape of the pocket for HMW-PAHs, but also increases the hydrophobicity afforded by F300, which is beneficial for aromatic substrates (Fig. 7*B*). Notably, NarA2B2^V236A-W316I-L375F^ demonstrated optimal degradation performance for CHR, likely due to F375 enhancing the π-π interactions between the substrate and the protein.

## Discussion

HMW-PAHs present a significant obstacle in environmental remediation due to their elevated toxicity and strong resistance to degradation (24). The RHO is an enzyme required for the initial step of aerobic microbial degradation of PAHs, determining the substrate spectrum of microorganisms (5, 19). However, currently, only a few RHOs capable of degrading HMW-PAHs have been reported (25). Modifying existing RHOs may potentially enhance microbial degradation capabilities towards HMW-PAHs. Rational design, as a common enzyme engineering strategy, has been successfully applied to the modification of various biological catalysts (15, 16, 17, 18).

NarA2B2 is an RHO in *Hydrogenibacillus* sp. N12, capable of catalyzing multiple LMW-PAHs (19). In this study, we employed structure-guided rational enzyme engineering to modify the RHO, endowing it with the capability to catalyze HMW-PAHs. The substrate specificity of the RHO is intricately linked to the shape and size of their active site pockets (26). These distinctive characteristics of the active site pocket are closely associated with the amino acids that contribute to their formation. Many of these amino acids possess hydrophobic properties, which aid in binding aromatic compounds within the active site pocket (27, 28, 29, 30). Key amino acid residues within the active site pocket can influence its dimensions, affect the orientation of substrates, and modify the regioselectivity of enzymes (31). For instance, in the RHO, PhnI, specific residues like F350, F404, and L356 play a significant role in shaping the active site pocket into a uniform trapezoidal configuration, enabling PhnI to efficiently degrade HMW-PAHs (31). Consequently, a few crucial amino acid residues within the active site can confer distinct catalytic properties upon the enzyme. The identification of key amino acid residues is the foundation for the rational design of enzymes (32). We have identified four “hotspot” residues (V236, Y300, W316, and L375) that appear to restrict the catalytic capacity of NarA2B2 to degrade HMW-PAHs, with comprehensive assessments of static (structure prediction and molecular docking) conformations. To unlock NarA2B2’s potential for catalyzing HMW-PAHs, we utilized structure-guided rational enzyme engineering to introduce specific amino acid substitutions. These substitutions effectively broadened NarA2B2’s ability to degrade a wider range of HMW-PAHs. For example, NarA2B2^W316I^ and NarA2B2^Y300F-W316I^ demonstrated exceptional degradation of FLU, PYE, BaA, BbF, and BaP, while NarA2B2^V236A-W316I-L375F^ also proved highly effective in catalyzing CHR. The modified NarA2B2 stands as the promising candidate for potential bioremediation applications.

The RHO is a multi-component enzyme, consisting of terminal oxygenase and electron transfer protein. RHOs require compatible electron transfer proteins to exert catalytic functions, thus different electron transfer proteins can also influence the catalytic efficiency of an RHO (33, 34). Specifically, the specific activity to convert PHE by NidA3B3 varied, reaching 0.15 ± 0.03 U/mg when adapted to PhtAcAd and 0.025 ± 0.006 U/mg when adapted to PhdCD (33). When the toluene dioxygenase in *Pseudomonas putida* F1 adapted various electron transfer proteins, a distinct difference in catalytic efficiency was observed (34). The investigation of the biochemical properties and catalytic activity of purified NarA2B2 and NarAaAb was initially conducted with the assumption of using PhtAcAd as the electron transfer protein. However, when detecting the catalytic activity of NarAaAb on PAHs using the resting cell biotransformation, the enzyme was found to require electron transfer using either PhtAcAd or PhdCD (20). To explore the influence of different electron transfer proteins on the biochemical properties, we purified PhdCD and examined the biochemical characteristics of NarAaAb adapted with either PhtAcAd or PhdCD. With PhdCD as the electron transfer protein, NarAaAb exhibited an optimal catalytic temperature of 42 °C, which is significantly higher than that observed with PhtAcAd (Fig. S5). Notably, NarAaAb retained 20% catalytic activity for PHE at pH six when adapted with PhdCD (Fig. S5). These findings indicated that the characteristics of an RHO differed depending on the specific electron transfer protein used. Additionally, through the thermal shift assay, we determined that the melting temperature (Tm) value for NarAaAb is 92.6 °C, while the Tm value for NarA2B2 is 85.3 °C (the data was not shown), indicating that both enzymes possess high thermal stability. Consequently, it can be speculated that if adapted to endogenous electron transfer proteins, NarA2B2 and NarAaAb could potentially catalyze PAHs at elevated temperatures.

Therefore, modifying existing enzymes and seeking compatible electron transfer proteins can effectively enhance the catalytic efficiency of RHOs towards HMW-PAHs, aiding in addressing HMW-PAHs pollution.

## Experimental procedures

### Chemicals and bacterial strains

PAHs and heterocyclic compounds were purchased from J&K Scientific Co, Ltd or Shanghai Aladdin Biochemical Technology Co, Ltd. The purity of all the chemicals surpassed 95%. PAHs and derivatives were dissolved using *N, N*-dimethylformamide as stock solutions and the appropriate volume was added to the sample before use. *Escherichia coli* (*E. coli*) Top10 and *E. coli* BL21(DE3) were used for plasmid construction or gene expression, respectively. *E. coli* was cultured at 37 °C in Luria-Bertani medium containing different antibiotics.

### Cloning and molecular biology experiments

Using the genomic DNA of *Hydrogenibacillus* sp. strain N12 as a template, the *narA2* and *narB2* genes were separately amplified. Subsequently, these genes were separately ligated into the two open reading frames (ORFs) of plasmid pETDuet, with a terminator inserted between ORF1 and ORF2, generating the plasmid pETDuet-*narA2*-Ter-*narB2*. The *narAa* gene, *narAb* gene, and the spacer sequence between them were amplified, and the DNA fragment was inserted into pET28a, creating plasmid pET28a-*narAaAb*.

Due to the absence of endogenous electron transfer proteins compatible with NarA2B2 and NarAaAb in *Hydrogenibacillus* sp. strain N12, exogenous electron transfer proteins, PhtAcAd (derived from *Mycobacterium vanbaalenii* PYR-1) (35) and PhdCD (derived from *Nocardioides* sp. strain KP7) (6), were introduced to investigate the catalytic activity of NarA2B2 and NarAaAb. The *phtAc* and *phdC* genes encode ferredoxins, while the *phtAd* and *phdD* genes encode ferredoxin reductases. The genes encoding the electron transfer proteins were synthesized following a search on the National Center for Biotechnology Information (NCBI). Additionally, the *phtAcAd* and *phdCD* genes were individually inserted into the ORF1 of plasmid pACYCDuet, generating plasmids pACYCDuet-*phtAcAd* and pACYCDuet-*phdCD*.

For the convenience of protein purification using Ni-NTA affinity chromatography, the *phtAcAd* and *phdCD* genes were also inserted into pET28a, resulting in plasmids pET28a-*phtAcAd* and pET28a-*phdCD*. Plasmid maps are shown in Fig. S6.

### Activity assays for NarA2B2 and NarAaAb

The catalytic activity assays for both the WT and variants of NarAaAb and NarA2B2 were conducted using both *in vivo* and *in vitro* methods.

*E. coli* BL21(DE3) cells harboring recombinant plasmids were cultured at 37 °C. When the OD_600nm_ reached 0.8, isopropyl-β-D-thiogalactopyranoside was added to induce protein expression, reaching a final concentration of 0.4 mM. Following 16 h of induction at 16 °C, the cells were harvested and washed three times with a phosphate-buffered saline solution. Thereafter, the cells were subjected to a three-hour starvation period without any substrates to prepare resting cells. We introduced PAHs or heterocyclic compounds into these resting cells to evaluate their degradation capacity. The final concentrations for LMW-PAHs and heterocyclic compounds were established at 50 mg/L (except for 40 mg/L for ANT), while HMW-PAHs were maintained at a final concentration of 10 mg/L. To determine the concentration of residual substrates in the samples, we extracted them with ethyl acetate and subsequently analyzed by HPLC (36). Specifically, HMW-PAHs were extracted with half a volume of ethyl acetate for 6 min, while LMW-PAHs were extracted with an equal volume of ethyl acetate for 3 min. *E. coli* BL21(DE3) strains containing empty plasmids were utilized as a control, following the same experimental procedures as the experimental group. All tests above were conducted in triplicate.

The *in vitro* catalytic activity assays employed purified proteins. The protein was purified using the same method as previously described for NarA2B2 (19). The total volume of the reaction mixture was 1 ml, containing 25 mM Tris-HCl pH 7, 10% glycerol, 0.05 mM ferrous ammonium sulfate, and 0.3 mM L-ascorbic acid. 5.67 μM of the RHO and 10 μM of the electron transfer protein were added to the reaction mixture, and the incubation was carried out under anaerobic conditions for 30 min. Subsequently, 0.2 mM NADH and the appropriate substrate were added under anaerobic conditions. After transferring the reaction mixture from an anaerobic environment to a laboratory setting, O_2_ from the air entered the reaction mixture, initiating the catalytic reaction. Following the catalytic reaction at 37 °C for 2 h, 20 μl of HCl was added to terminate the reaction. Subsequently, half the volume of ethyl acetate was added to extract the substrate or product for subsequent detection and analysis.

The degradation percentage of PAHs was calculated as the percentage decrease in substrate concentration in the experimental group minus the percentage decrease in substrate concentration in the control group. The calculation for the percentage decrease in substrate concentration was determined by subtracting the substrate concentration of the 24-h sample from that of the 0-h sample and then dividing this difference by the substrate concentration of the 0-h sample (19).

### Determination of metabolites by gas chromatography-mass spectrometry

Product extraction and detection methods were conducted similarly to those previously described (36). The metabolites of HMW-PAHs catalyzed by modified NarA2B2 were detected through gas chromatography-mass spectrometry (GC-MS). The degradation samples of HMW-PAHs obtained through resting cell biotransformation and enzyme catalysis were subjected to extraction with half volume of ethyl acetate for 6 min. The organic extract was then separated, and HCl was added to the remaining sample to adjust the pH to 1 to 2. Subsequently, half a volume of ethyl acetate was added for another 6 min of extraction. The organic extract from the two extractions was combined and dried with anhydrous sodium sulfate to remove residual water. After concentrating the sample 50 times, 30 μl of the sample was mixed with an equal volume of *N*,*O*-bis(trimethylsilyl)trifluoroacetamide and derivatized at 70 °C for 30 min. Detection was then performed using GC-MS (Agilent & GC-7890B; MS-5977B; 30 m × 0.25 mm, 0.25 μm, HP-5MS column). The GC-MS oven temperature was initially set at 75 °C for 3 min, then ramped up to 250 °C at a rate of 12 °C/min, where it was maintained for 1 min. Subsequently, the temperature was increased to 300 °C at a rate of 10 °C/min and held at 300 °C for 10 min.

### Molecular docking

The structures of NarAaAb and NarA2B2 were predicted using AlphaFold2 Multimer (37). Molecular docking with AutoDock 4.2 was used to predict the binding modes of substrates (38). The ligand coordinates were acquired from the PubChem database and prepared with GaussView 6 (39). AutoDockTools 1.5.6 was used to prepare the protein and substrate PDBQT files, adding polar hydrogens, removing water molecules, assigning Gasteiger charges, and defining rotatable bonds for the ligands. AutoDockTools 1.5.6 was also used to generate the grid parameter files for the receptor, with a grid size of 60 × 60 × 60 Å, centered on the active site. The docking calculations were performed with AutoDock 4.2, using the Genetic Algorithm with default parameters and establishing the number of modes as 100. The optimal ligand conformations were selected according to the binding affinity and the appropriate attack site.

### Point mutants

To create point mutants, primers were designed with a nine-bp overlap containing the mutation site. The WT plasmid served as the template. PCR products were digested using *Dpn*I. Positive transformants were examined through PCR and sequencing. Primers for the construction of mutants are listed in Table S3.

## Conclusion

NarAaAb and NarA2B2, both derived from *Hydrogenibacillus* sp. strain N12, shared highly similar secondary structures. However, their substrate specificities diverged significantly. A comparison of their three-dimensional structures allowed us to pinpoint the “hotspot” residues influencing NarA2B2’s catalysis of HMW-PAHs. Utilizing structure-guided rational enzyme engineering, we successfully modified NarA2B2, conferring its catalytic proficiency for six types of HMW-PAHs. Computational biology analysis revealed alterations in the size and hydrophobicity of the active site pocket of the modified NarA2B2, facilitating the entry of HMW-PAHs into the active site pocket.

## Data availability

The whole-genome sequence of *Hydrogenibacillus* sp. strain N12 was deposited into the NCBI database under the accession number CP080953. The protein IDs of NarAa and NarAb were QZA33825.1 and QZA33826.1. The protein ID of NarA2 was QZA33833.1. The protein IDs of PhtAc and PhtAd were AAQ91918.1 and AAQ91919.1. The protein IDs of PhdC and PhdD were BAA94713.1 and BAA94714.1.

## Supporting information

This article contains supporting information (21).

## Conflict of interest

The authors declare no competing interests.
