# Supplementary material for: Fine-tuning an aromatic ring-hydroxylating oxygenase to degrade high molecular weight polycyclic aromatic hydrocarbon
Source: J Biol Chem. 2024 May 4;300(6):107343. doi: 10.1016/j.jbc.2024.107343 (PMC11176777; doi:10.1016/j.jbc.2024.107343)
Supplement: Supplemental Figures S1–S6 and Tables S1–S3 [file mmc1.pdf]

TITLE:

Fine-tuning an aromatic ring-hydroxylating oxygenase to degrade high molecular weight polycyclic aromatic hydrocarbon

Lihua Guo†, Xingyu Ouyang†, Weiwei Wang, Xiaoyu Qiu, Yi-Lei Zhao, Ping Xu and Hongzhi Tang*

State Key Laboratory of Microbial Metabolism, and School of Life Sciences & Biotechnology, Shanghai Jiao Tong University, Shanghai 200240, People's Republic of China

†These authors contributed equally to this study.

*Corresponding author: H. Z. Tang

Mailing address: School of Life Sciences & Biotechnology, Shanghai Jiao Tong University, Shanghai 200240, P. R. China

E-mail: tanghongzhi@sjtu.edu.cn; Tel: +86-21-34204066; Fax: +86-21-34206723.

Figures S1-S6

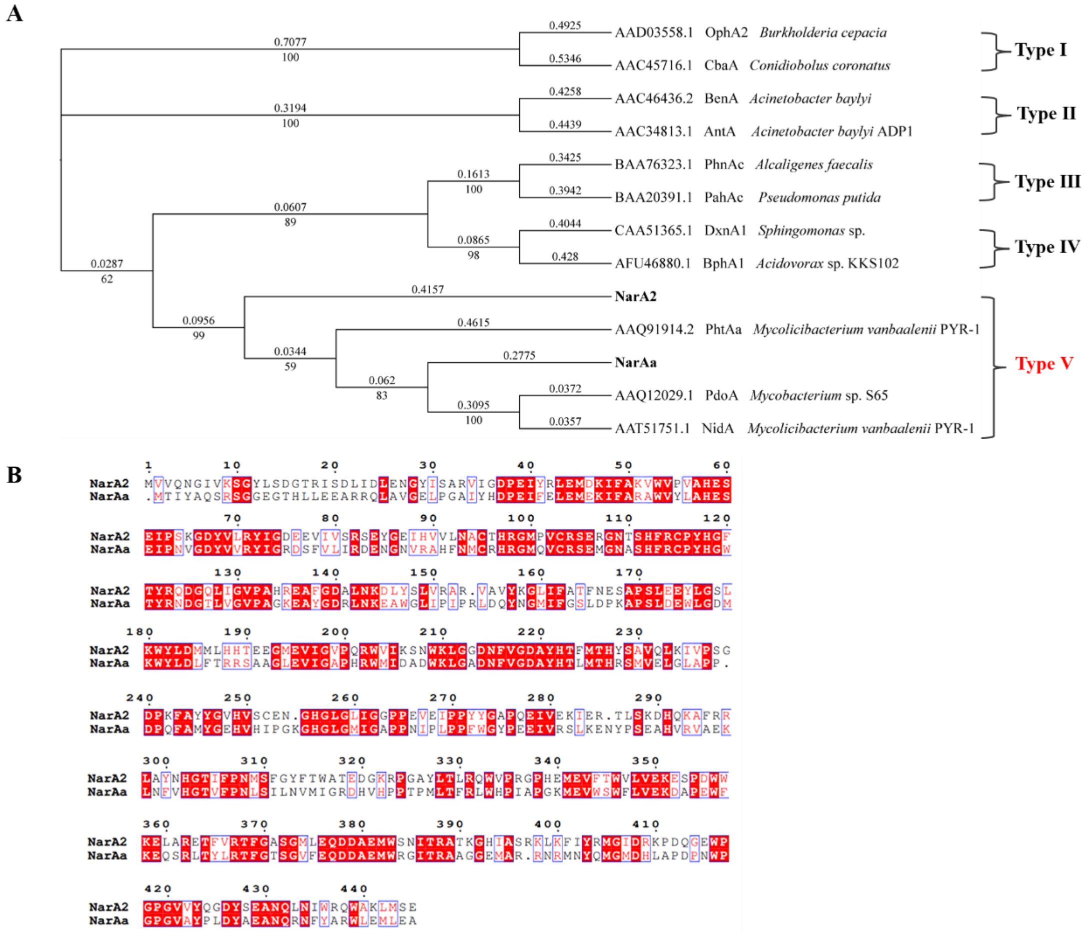

Figure S1. The phylogenetic tree and sequence similarity analysis of NarA2 and NarAa, the α subunits of NarA2B2 and NarAaAb, respectively. (A) Neighbor-joining phylogenetic tree of amino acid sequences of NarA2 and NarAa. The phylogenetic tree was constructed based on a novel classification system for aromatic ring-hydroxylating oxygenases, which categorized them into five types[21]. (B) The sequence alignment results of NarAa and NarA2 by ENDscript.

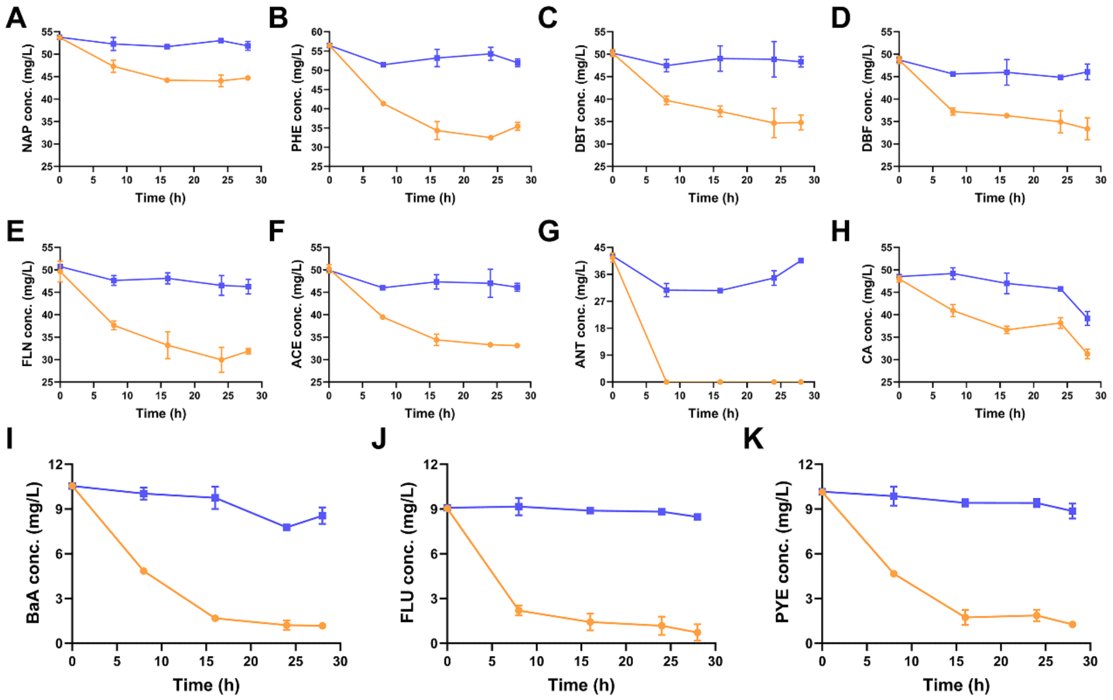

Figure S2. The degradation of PAHs and derivatives by resting cells with heterologous expression of NarAaAb and PhdCD. (A) NAP (50 mg/L); (B) PHE (50 mg/L); (C) DBT (50 mg/L); (D) DBF (50 mg/L); (E) FLN (50 mg/L); (F) ACE (50 mg/L); (G) ANT (40 mg/L); (H) CA (50 mg/L); (I) BaA (10 mg/L); (J) FLU (10 mg/L); (K) PYE (10 mg/L). The yellow line with circles represents *E. coli* BL21(DE3) containing pET28a-*narAaAb* and pACYCDuet-*phdCD*, and the blue line with squares represents *E. coli* BL21(DE3) containing pET28a and pACYCDuet. The concentration in parentheses is the initial concentration. NAP, naphthalene; PHE, phenanthrene; DBT, dibenzothiophene; DBF, dibenzofuran; FLN, fluorene; ACE, acenaphthene; ANT, anthracene; CA, carbazole; BaA, benzo[a]anthracene; FLU, fluoranthene; PYE, pyrene.

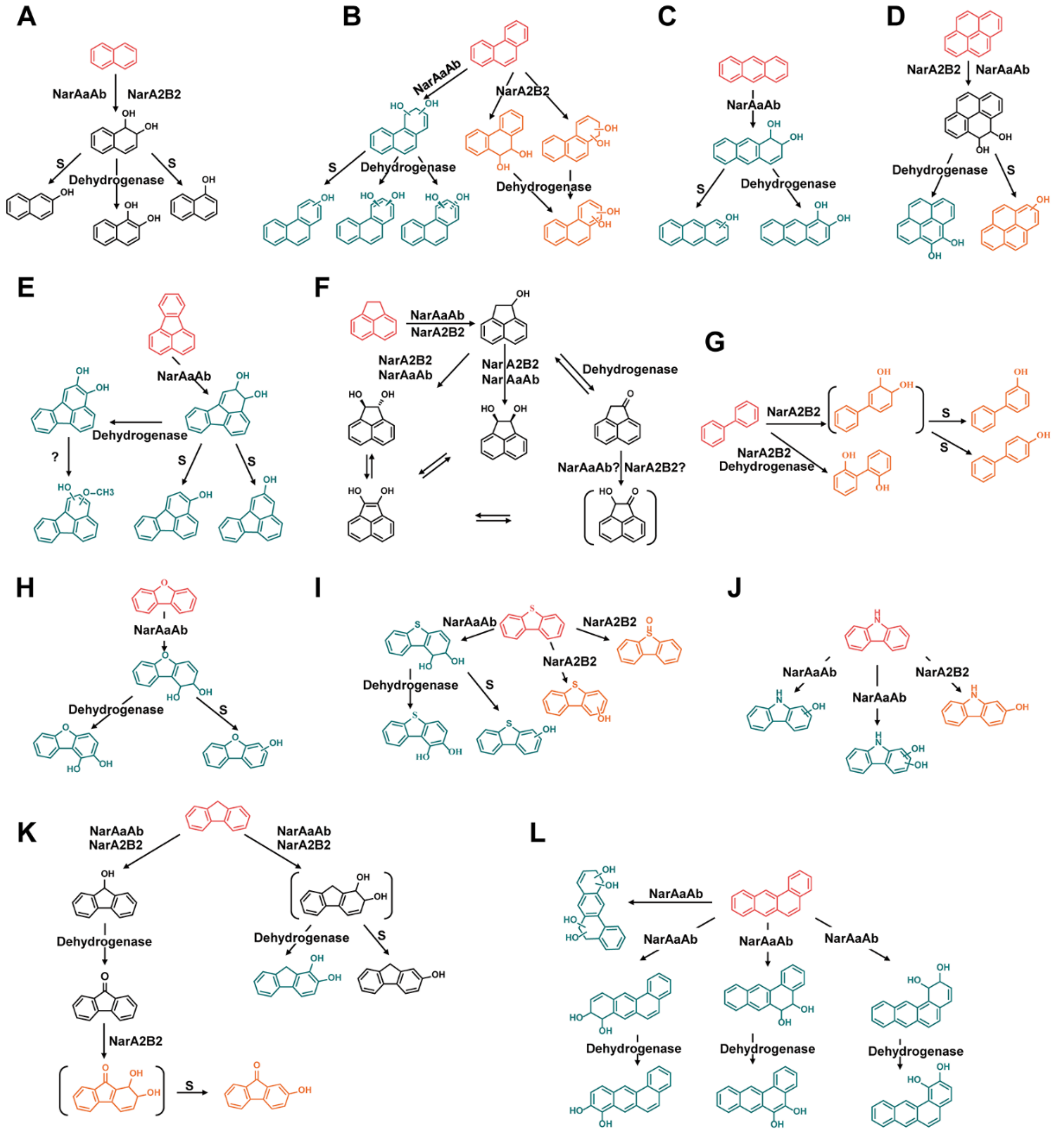

Figure S3. The metabolic characteristics of PAHs and its derivatives catalyzed by NarA2B2 and NarAaAb. The red compounds represent the substrate. The black compounds were produced by NarAaAb and NarA2B2. While the green and orange compounds were only produced by NarAaAb and NarA2B2, respectively. The metabolites in parentheses were inferred from the reported pathway and were not detected in this study. (A) naphthalene, NAP; (B) phenanthrene, PHE; (C) anthracene, ANT; (D) pyrene, PYE; (E) fluoranthene, FLU; (F) acenaphthene, ACE; (G) biphenyl, BP; (H) dibenzofuran, DBF; (I) dibenzothiophene, DBT; (J) carbazole, CA; (K) fluorene, FLN; (L) benzo[a]anthracene, BaA.

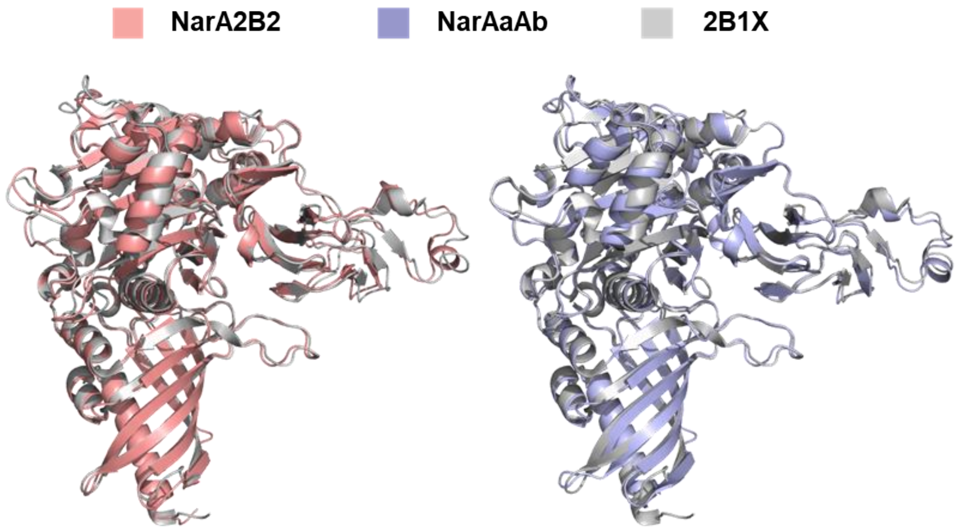

Figure S4. Structure comparing of NarAaAb and NarA2B2 with their homolog structure, the naphthalene 1,2-dioxygenase from *Rhodococcus* sp. (PDB ID: 2B1X).

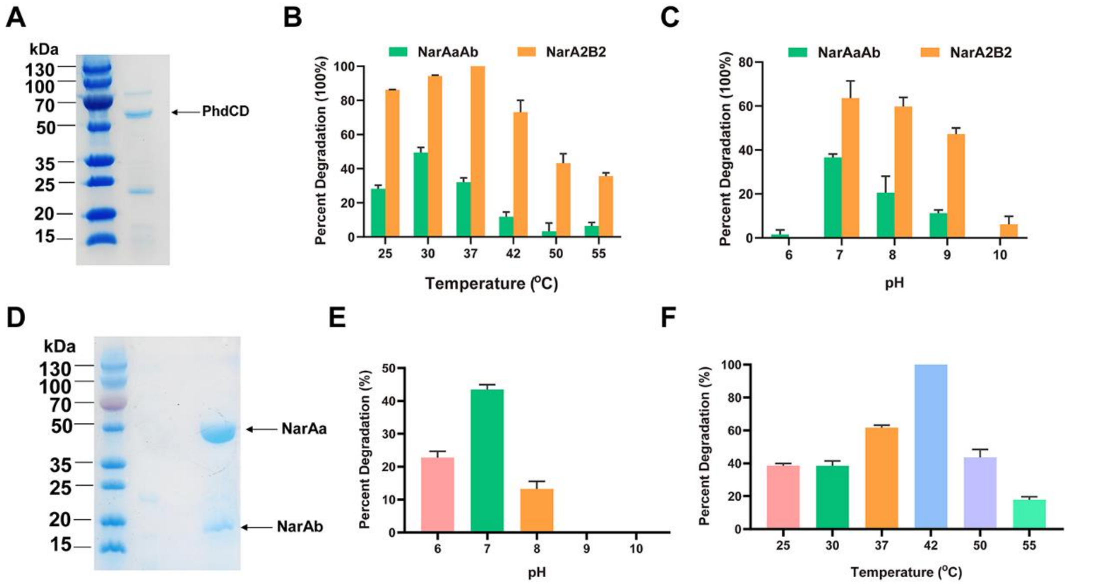

Figure S5. The biochemical properties of purified NarAaAb and NarA2B2. (A) The purification of the electron transport protein PhdCD. (B) The catalytic efficiency of purified NarAaAb and NarA2B2 at different temperatures when PhtAcAd was used as electron transport protein. (C) The catalytic efficiency of purified NarAaAb and NarA2B2 at different pH values when PhtAcAd was used as electron transport protein. (D) The purification of NarAaAb by Ni-NTA affinity chromatography. (E) The catalytic pH range suitable for NarAaAb when PhdCD was used as electron transport protein. (F) The catalytic temperature range suitable for NarAaAb when PhdCD was used as electron transport protein.

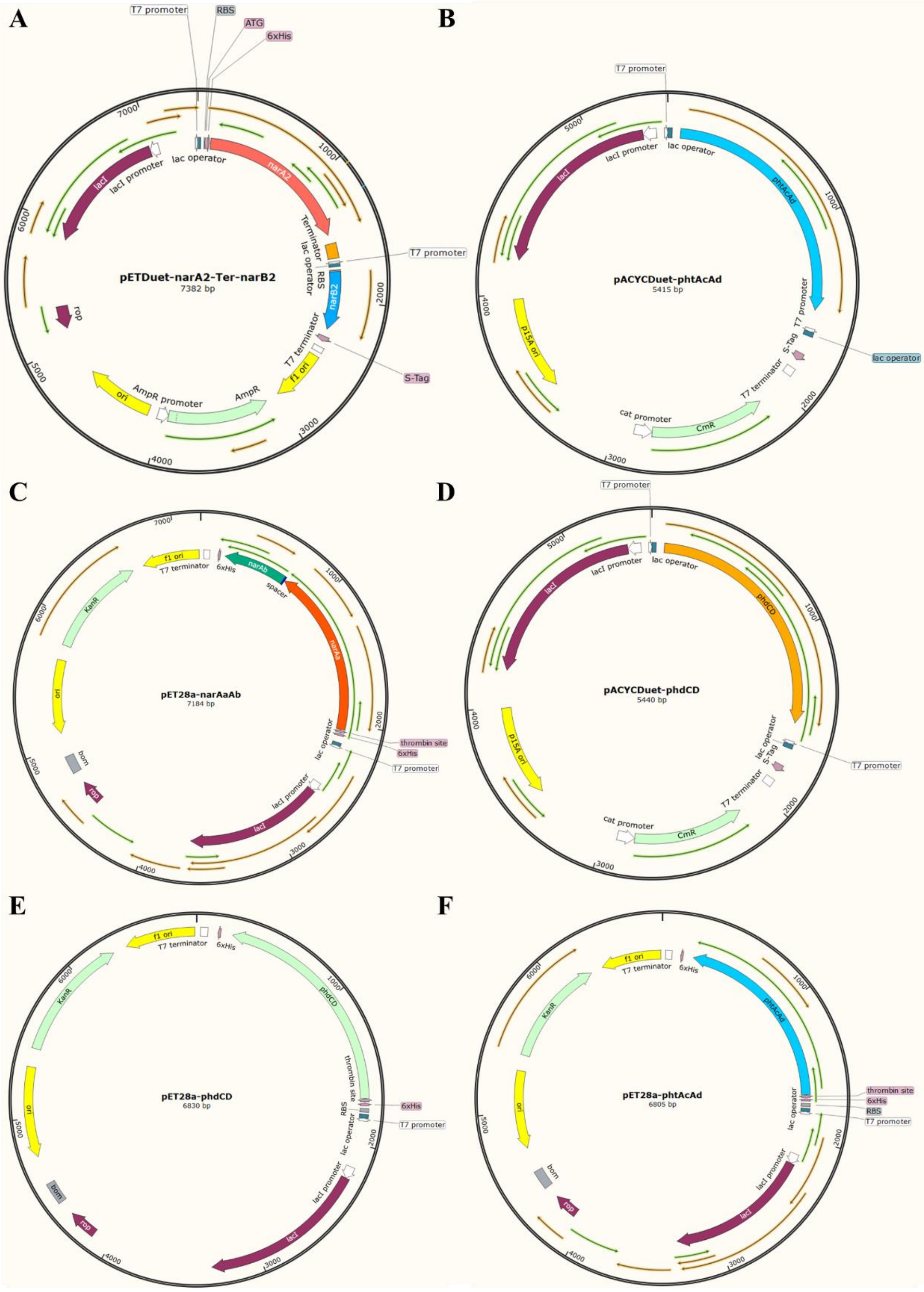

Figure S6. The plasmid maps of the expression vectors for the aromatic ring-hydroxylating oxygenases and electron transfer proteins used in this study. (A) pETDuet-*narA2*-Ter-*narB2*. (B) pACYCDuet-*phtAcAd*. (C) pET28a-*narAaAb*. (D) pACYCDuet-*phdCD*. (E) pET28a-*phdCD*. (F) pET28a-*phtAcAd*.

Table S1. GC-MS analysis of PAHs metabolites catalyzed by NarAaAb.

Substrate	Proposed product	Structure	Retention Time (min)	Mass spectral characteristics of product (ion abundances)
NAP	1-naphthol (TMS)	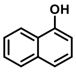	12.281	216 (100), 201(97), 185(48), 73(30), 217(19), 202(18), 115(18)
	2-naphthol (TMS)	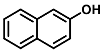	12.536	201(100), 216(85), 73(48), 71(29), 57(25), 145(24), 75(23)
	NAP-1,2-dihydrodiol (2TMS)	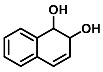	13.431	191(100), 73(88), 147(60), 203(32), 128(23), 192(22), 306(18)
	1,2-dihydroxynaphthalene (2TMS)	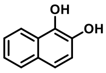	14.597	73(100), 304(64), 216(22), 55(19), 305(17), 70(17), 83(14)
PHE	monohydroxy-PHE (TMS)	—	16.856	266(100), 235(69), 251(59), 73(30), 267(24), 67(16)
	PHE dihydrodiol (2TMS)	—	17.387	191(100), 73(72), 147(39), 356(28), 192(22), 253(22)
	dihydroxy-PHE (2TMS) (trans or cis)	—	18.213	73(100), 354(76), 266(25), 355(25), 236(17), 356(9)
	dihydroxy-PHE (2TMS) (trans or cis)	—	18.652	354(100), 73(79), 355(32), 75(17), 57(12), 356(12)
ANT	monohydroxy-ANT (TMS)	—	17.277	266(100), 73(32), 251(31), 267(23), 235(21), 165(13)
	ANT-1,2-dihydrodiol (2TMS)	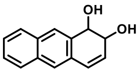	17.641	191(100), 73(73), 253(64), 147(37), 165(21), 192(21)
	1, 2-dihydroxy-ANT (2TMS)	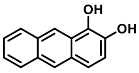	19.183	354(100), 73(64), 355(32), 266(27), 236(16), 356(12)
FLN	9-fluorenone	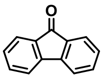	14.361	180(100), 152(38), 151(22), 181(15), 150(13), 76(13)
	2-fluoreonl (TMS)	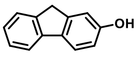	15.822	254(100), 223(100),239(63), 255(31), 224(27), 165(19)
	9-fluorenol (TMS)	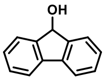	16.139	254(100), 239(82), 165(66), 255(27), 240(20), 73(17)
	1,2-dihydroxy-FLN (2TMS)	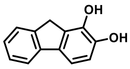	17.618	73(100), 342(99), 253(71), 343(39), 254(34), 223(18)
ACE	1-acenaphthenone	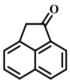	13.622	140(100), 168(96), 139(85), 70(15), 169(14), 141(13)
	1-acenaphthenol (TMS)	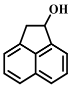	14.517	242(100), 75(91), 152(88), 241(76), 153(63), 243(36)
	1,2-dihydroxyacenaphthene (2TMS) (trans or cis)	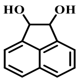	16.168	147(100), 73(74), 330(33), 168(33), 241(25), 315(21)
	1,2-dihydroxyacenaphthene	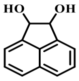	16.088	147(100), 73(55), 330(48), 168(37), 315(36), 241(32)

	(2TMS) (trans or cis)			
	1,2-dihydroxyacenaphthylene (2TMS)	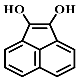	16.631	73(100), 328(91), 311(43), 75(36), 117(33), 240(28)
DBT	monohydroxy-DBT (TMS)	—	17.179	272(100), 241(65), 257(54), 202(36), 73(22), 272(22)
	DBT-1,2-dihydrodiol (2TMS)	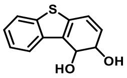	17.531	272(100), 257(86), 362(61), 73(59), 273(33), 147(31)
	1,2-dihydroxy-DBT (2TMS)	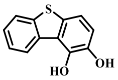	18.946	73(100), 360(70), 272(23), 361(22), 242(18), 362(12)
DBF	monohydroxy-DBF (TMS)	—	15.631	241(100), 256(100), 242(40), 257(33), 139(24), 181(21)
	monohydroxy-DBF (TMS)	—	15.799	241(100), 256(98), 257(21), 73(21), 242(21), 254(15)
	1,2-dihydroxy-DBF (2TMS) (cis or trans)	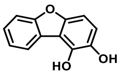	17.179	73(100), 344(57), 345(17), 226(12), 256(12), 74(9)
	1,2-dihydroxy-DBF (2TMS) (cis or trans)	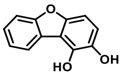	17.277	73(100), 344(64), 345(20), 256(19), 226(15), 74(9)
	DBF-1,2-dihydrodiol (2TMS)	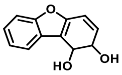	16.272	346(100), 73(70), 147(45), 347(42), 156(27), 168(22)
CA	monohydroxy-CA (TMS)	—	17.075	255(100), 239(61), 240(58), 224(58), 209(30), 256(26)
	dihydroxy-CA (2TMS)	—	20.765	343(100), 344(30), 73(25), 328(18), 345(11), 329(6)
PYE	PYE-4,5-dihydrodiol (2TMS)	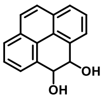	19.328	147(100), 290(78), 291(50), 73(47), 380(43), 365(36)
	4,5-dihydroxy-PYE (2TMS)	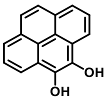	21.222	378(100), 73(79), 290(44), 379(35), 260(20), 380(13)
FLU	FLU-2,3-dihydrodiol (2TMS)	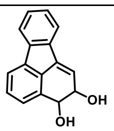	20.096	73(100), 147(80), 380(68), 190(57), 218(38), 291(32)
	monohydroxy-FLU (TMS)	—	19.905	290(100), 259(57), 275(51), 291(28), 73(26), 130(16)
	monohydroxy-FLU (TMS)	—	20.315	290(100), 275(75), 291(31), 276(21), 189(21), 200(19)
	2,3-dihydroxy-FLU (2TMS)	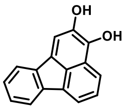	21.857	73(100), 378(100), 379(37), 290(27), 260(26), 380(14)
	x-methoxy-y-hydroxy-FLU (TMS)	—	21.158	290(100), 320(62), 291(26), 260(20), 73(17), 321(17)

	BaA-5,6-dihydrodiol (2TMS)	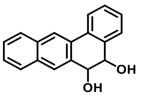	23.018	316(100), 317(29), 73(24), 301(23), 226(16), 406(3)
	BaA-8,9-dihydrodiol (2TMS)	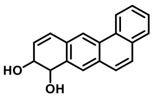	22.556	191(100), 73(91), 303(58), 147(34) ,406(23), 215(23)
BaA	BaA-1,2-dihydrodiol (2TMS)	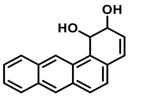	22.284	191(100), 73(81), 406(47), 147(31), 303(27), 228(26)
	dihydroxy-BaA (2TMS)	__	23.289	404(100), 73(80), 405(37), 207(24), 57(13), 147(13)
	dihydroxy-BaA (2TMS)	__	23.382	404(100), 73(95), 405(36), 316(30), 286(19), 406(13)
	dihydroxy-BaA (2TMS)	__	24.306	404(100), 73(92), 207(53), 405(40), 316(25), 286(17)
	BaA-bis-dihydrodiol (4TMS)	__	24.023	191(100), 73(86), 147(25), 192(18), 481(4.5), 148(4)

TMS: trimethylsilyl derivatized compound; 2TMS: ditrimethylsilyl derivatized compound.

The metabolites whose structure was not determined were indicated by "__".

Table S2. The yield of catalytic products of HMW-PAHs by the modified NarA2B2.

Substrate	Variant	Product	Yield
BaA	$NarA2B2^{W316I}$	BaA-1,2-dihydrodiol	6.61%
	$NarA2B2^{Y300FW316I}$	BaA-8,9-dihydrodiol	0.14%
	$NarA2B2^{Y300FW316I}$	BaA-1,2-dihydrodiol	4.40%
PYE	$NarA2B2^{Y300FW316I}$	PYE-4,5-dihydrodiol	6.86%
FLU	$NarA2B2^{W316I}$	FLU-2,3-dihydrodiol	3.28%
	$NarA2B2^{Y300FW316I}$	FLU-2,3-dihydrodiol	7.29%
CHR	$NarA2B2^{V236AW316IL375F}$	CHR-3,4-dihydrodiol	0.03%
BaP	$NarA2B2^{Y300FW316I}$	BaP-9,10-dihydrodiol	0.11%
	$NarA2B2^{Y300FW316I}$	BaP-dihydrodiol	0.03%
BbF	$NarA2B2^{W316I}$	BbF-dihydrodiol	0.06%
	$NarA2B2^{W316I}$	BbF-dihydrodiol	0.08%

Table S3. Primers used in the construction of variants.

Primer	Sequence
NarA2B2^{V236A}-F	ATCGCACCGAGCGGCGATCCA
NarA2B2^{V236A}-R	CGGTGCGATCTTCAACTGGACAGCAGA
NarA2B2^{Y300F}-F	GCATTTAACCATGGCACCATC
NarA2B2^{Y300F}-R	GTTAAATGCCAGACGCCGGAA
NarA2B2^{W316I}-F	ACCATAGCCACGGAAGATGGC
NarA2B2^{W316I}-R	GGCTATGGTGAAGTAACCGAACGACAT
NarA2B2^{L375F}-F	ATGTTTGAACAGGACGATGCG
NarA2B2^{L375F}-R	TTCAAACATACCGCTCGCACC
NarAaAb^{I317A}-F	ATGGCAGGCCGCGATCACGTCCAT
NarAaAb^{I317A}-R	GCCTGCCATGACGTTAAGGATGGAAAGATTCGGAAACACC
NarAaAb^{F376A}-F	GTGGCAGAGCAGGACGACGCGGAA
NarAaAb^{F376A}-R	CTCTGCCACCCCGCTCGTCCCGAA
NarAaAb^{A236G}-F	CTTGGACCCCCAGATCCGCAATTT
NarAaAb^{A236G}-R	GGGTCCAAGCCCAAGTTCGACCAT
NarAaAb^{F301A}-F	AACGCAGTCCATGGAACGGTGTTT
NarAaAb^{F301A}-R	GACTGCGTTCAACTTTTCGGCGAC
